# Do Emergency Physicians and Medical Students Find It Unethical to ‘Look up’ Their Patients on Facebook or Google?

**DOI:** 10.5811/westjem.2015.1.24258

**Published:** 2015-02-25

**Authors:** Maxim Ben-Yakov, Ahmed Kayssi, Jennifer D. Bernardo, Christopher M. Hicks, Karen Devon

**Affiliations:** * University of Toronto, Department of Emergency Medicine, Toronto, Ontario; † University of Toronto, Department of Surgery, Toronto, Ontario

## Abstract

**Introduction:**

The use of search engines and online social media (OSM) websites by healthcare providers is increasing and may even be used to search for patient information. This raises several ethical issues. The objective of this study is to evaluate the prevalence of OSM and web-searching for patient information and to explore attitudes towards the ethical appropriateness of these practices by physicians and trainees in the emergency department (ED).

**Methods:**

We conducted an online survey study of Canadian emergency physicians and trainees listed under then Canadian Association of Emergency Physicians (CAEP) and senior medical students at the University of Toronto.

**Results:**

We received 530 responses (response rate 49.1%): 34.9% medical students, 15.5% residents, 49.6% staff physicians. Most had an active Facebook account (74%). Sixty-four participants (13.5%) had used Google to research a patient and 10 (2.1%) had searched for patients on Facebook. There were no differences in these results based on level of training, and 25% of physicians considered using Facebook to learn about a patient “very unethical.” The most frequent ethical concerns were with violation of patient confidentiality, dignity, and consent. The practice was usually not disclosed to patients (14%), but often disclosed to senior colleagues (83%).

**Conclusion:**

This is the first study examining the prevalence of and attitudes towards online searching for obtaining patient information in the ED. This practice occurs among staff physicians and trainees despite ethical concerns. Future work should explore the utility and desirability of searching for patient information online.

## INTRODUCTION

Search engines and social media websites, such as Facebook, are being used increasingly in professional, social, and political arenas. The frequency of Facebook use by physicians is comparable to that of the general public, with at least 53% subscribing.^1^,^2^

In the emergency department (ED), key past medical history and demographic information can be missing, particularly in new, unidentified or non-communicative patients. This may contribute to diagnostic uncertainty, treatment errors and delays in disposition, and may increase the likelihood of adverse events. Online social media (OSM) offers users an unprecedented opportunity to share and access personal health information,^3^ which can be searched for and accessed online by health professionals when it is not available by more traditional means of obtaining a medical history. However, the intersection of private health information with public online tools poses risks to medical professionalism. Recent major popular media outlets highlight the importance of balancing the need for urgent information (that could be obtained via OSM or search engines) versus the need to maintain patient privacy and public trust.^4^,^5^ Although the volume of personal information accessible online is increasing, the boundaries of the digital doctor-patient relationship have not yet been defined.^1^,^6^

Recently medical authorities have published guidelines discussing OSM use, primarily addressing interactions directly with patients and online posting of patient results.^2^,^6^–^13^ The American College of Physicians^12^ and the American Heart Association (AHA)^11^ guidelines highlight principles of online professionalism. The AHA primarily discourages the practice of searching for patients online, unless it is used to advance patient care (such as in emergency situations). However, the prevalence of this practice in the ED is currently unknown.

The goals of this survey study are to 1) establish the prevalence of OSM and web searching of patient information by emergency physicians and trainees and to determine whether the searches were disclosed to patients or senior preceptors; 2) determine the variables associated with the likelihood of looking-up a patient using Facebook; and 3) explore attitudes regarding the ethical appropriateness of these practices.

## METHODS

### Survey Development

We based survey development on a formal review of OSM literature in healthcare. A 25-item questionnaire was then generated using a commercially available online survey tool, available at SurveyMonkey.com. (SurveyMonkey Inc, Palo Alto, California, USA) We piloted the survey among 40 participants (including residents and staff) at one of our academic EDs. Key demographic data included age, gender, residency program and level of training. Respondents were asked about their use of electronic resources and OSM within the previous year, and the frequency with which they have used Facebook and Google to “search” for patients in the ED. Finally, respondents were surveyed on their views regarding the ethicality of online searching for patient information.

### Survey Distribution

The survey was distributed between November 2010 and June 2011 to 683 staff physicians, 116 residents and 54 medical students using the Canadian Association of Emergency Physicians (CAEP) email list, as well as to 226 fourth-year medical students at the University of Toronto. The survey was deployed using a modified Dilman approach:^14^ an introductory email was sent to potential respondents followed by the survey link and a reminder email two weeks later. Consent was implied by participation and after reading information provided by the investigators on Page 1 of the online survey. The study was approved by the Research Ethics Board at St. Michael’s Hospital and the University of Toronto Undergraduate Medical Education Office in Toronto, Canada.

### Data Analysis

Responses were automatically compiled and generated into Excel 2007 (Microsoft, Inc. Redmond, WA) as data tables by the SurveyMonkey tool (SurveyMonkey Inc., Palo Alto, CA). We then stripped these tables of any identifiers and each participant was assigned a unique ID number. Descriptive statistics were generated for all categorical data. We calculated chi-square test statistics for all binary and categorical data, and a p-value of <0.05 was considered significant. Uni- and multivariable-adjusted logistic regression models were used to assess the odds of using Facebook in the ED and a post-hoc analysis to determine the odds of searching a patient on Google. Variables included staff-status; age; gender; use of electronic patient record in the past year; and self-reported ethical objections to using OSM to research patients in the ED. We ascertained respondents’ locations via IP address geo-mapping using an online tool (batchgeo.com, BatchGeo LLC.). Statistical analysis was carried out using SAS 9.3 (Cary, North Carolina). For the multivariable adjusted logistic regression we incorporated all available data points and responses without excluding respondents who had missing data in their surveys.

## RESULTS

Of the 1,079 physicians and trainees surveyed, 530 responded (response rate of 49.1%). Forty-nine percent of those who responded were staff, 34.9% were medical students, and 15.5% were residents. Age, gender, program and level of training of the respondents are summarized in Table 1. Current use of social media by respondents is summarized in Table 2. Responses came from across Canada, including Ontario (47%), Manitoba (19%), British Columbia (8%), Alberta (7%) Quebec (5%), Eastern provinces (6%), Northern Territories and Saskatchewan (2%), as well as from international locations (6%). The majority of respondents (67.7%) had used an electronic medical record system for patient care in the ED during the past year.

Three-hundred and ninety-two (74%) respondents had a Facebook account, 102 (19.2%) had a Twitter account, 88 (16.6%) were on LinkedIn, and 98 (18.5%) reported having had a personal blog in the past year.

Sixty-four participants (12.1%) reported having used Google and 10 (1.9%) Facebook to search for patients while investigating them in the ED in the previous year. Patients were characterized as medical (43.1%), undifferentiated (33.3%), psychiatric (20.8%) or surgical (2.7%). As part of additional expletory questions, we queried respondents if they disclosed such a search to either patients or supervising senior physicians. Among those who used Google or Facebook to search for patients, only four (13%) disclosed that action to their patient. However, 29 (83%) trainees disclosed this information to their staff or senior resident.

On a Likert scale of one to seven (from very unethical to very ethical), among 435 replies to that question, 130 (29.8%) physicians and trainees considered the use of Facebook in this context “very unethical.” Eighteen (3.5%) respondents considered this practice “very ethical” (Figure). When asked about the ethical values breached by Google searching, respondents chose the following: confidentiality 184 (34.7%), informed consent 157 (29.6%), and patient dignity 124 (23.4%). Similarly, researching patients on Facebook was viewed as violating patient confidentiality 195 (36.8%), informed consent 168 (31.7%), and dignity 130 (24.5%).

A multivariable logistic regression demonstrated that there was no significant difference between staff and trainees in the likelihood of researching a patient on Facebook or on Google. The only statistically significant predictor of Facebook use was the belief that looking up a patient is “neutral, ethical or very ethical.” Those who responded that it was ethical or very ethical to use Facebook to research a patient were 10.37 (95% CI 1.28, 84.15) times more likely to do so than those who did not believe that it was ethical (Table 3). Similar results were obtained for the likelihood of searching patients on Google (Table 4).

## DISCUSSION

When faced with a paucity of patient data, especially in an unresponsive or uncooperative patient, EPs or trainees may explore several routes of obtaining patient information. Searching for patient information online may be a more modern version of searching through a patient’s wallet for information when they present with an altered level of consciousness. Although an online search will only reveal publically accessible information, it may nonetheless cause problems with respect to patient consent and accuracy of information.

Our results are consistent with previous reports that many physicians and healthcare professionals use Facebook, Twitter, and LinkedIn.^1^,^2^,^6^,^9^,^11^,^12^,^15^,^16^ With the exception of case reports,^17^ the practice of online searching for patient information has not been studied. This is the first study to examine the prevalence of physician and trainee use of Google or Facebook to search for patients who have presented to the ED. We demonstrated that while the practice is not very common, it does occur. Most existing studies citing breaches of privacy using social media, involve trainees.^3^,^16^,^18^–^25^ We found that the practice of searching for patients occurred equally among trainees and staff physicians. This held true even when we accounted for variations in non-professional use of social media by individuals. Based on our demographics, wide geographical coverage and fair response rate, we believe that our results are generalizable.

The behaviour was not usually disclosed to patients, although trainees sometimes disclosed this practice to senior colleagues. This raises questions about whether those engaging in the practice of online searches felt it was either questionable behavior and therefore not disclosed vs. unnecessary to disclose to patients since information was publicly available. Future scholarship should clarify this aspect.

The ethics and professionalism issues surrounding the use of OSM in medicine must be viewed from a multitude of perspectives.^11^ Our study highlights ethical concerns encountered by physicians attempting to learn more about their patients using online tools. When Bosslet et al*.* (2011) surveyed a general population of physicians through the American Medical Association (AMA), their results showed that 58% of physicians considered it unethical to view an online patient profile or communicate via OSM.^1^ In another study that used hypothetical vignettes to explore the same practice, faculty found the practice to be unethical, while trainees were open to using social media if it would help clinical care.^16^

Our results show that 29% of emergency medicine providers regarded searching for patients via Facebook to be “very unethical.” Those who felt it was ethical were significantly more likely to engage in the practice, which highlights the controversial nature of searching online for patients, rather than suggesting that physicians and trainees acted unethically. Even though information available on the Internet is public, respondents felt there could be a breach of confidentiality. They also felt that searching for patients could violate a patient’s dignity – implying that the issue goes beyond simply what information is publically available, to what information a patient would want his healthcare provider to know. Finally, informed consent was an ethical concern for respondents suggesting that perhaps a proportion of these searches were done in patients who had decisional capacity, although we do not have data to determine in which patients the searched were performed.

Although OSM offers many opportunities for knowledge dissemination, collaboration, education and interaction with the public, professional authorities and medical associations have cautioned physicians and trainees against its use and provided guidelines.^2^,^26^–^29^ The American College of Physicians (ACP)^12^ policy statement mentions both potential benefits and pitfalls of social media use. Benefits include observing and counselling patients on health-related behaviors, and intervening in emergency situations. Pitfalls include threatening the trust in the doctor-patient relationship and obtaining inaccurate sources of information.^12^ The AMA addresses the issue of posting confidential information online; however, they do not discuss that of accessing patient information online.^29^ Three provincial regulatory bodies in Canada have social media policies and all caution against the use of OSM or any form of online patient interaction.^13^,^17^,^26^,^27^ Searching online for patient information may be morally justifiable when it is done with the intent of enhancing patient care. However, healthcare professionals need to be cautious about “voyeuristic” searching for patients.^11^,^12^ Prior to engaging in an online search for patient information, healthcare professionals should thoughtfully consider the possible implications and accuracy of findings.^11^,^12^ We believe that when consent may not be obtained in the obtunded or uncooperative patient, when possible an online search should be disclosed to the patient in order to maintain the trust of the individual patient as well as public trust in general. Future research should further clarify patients’ perspectives on using online social media to search for information about them.

## LIMITATIONS

Our study is limited by several factors. The survey did not question whether physicians used Google+, LinkedIn, Twitter or other platforms to research patient information in order to limit the length of the survey. We also did not ascertain whether patients who were searched for online presented with an altered level of consciousness or inability to communicate or the type of information that was sought. The usefulness of information sought in the online search was difficult to ascertain due to a low response rate to that question in the survey.

EPs who were not members of CAEP were omitted from our study population. It is possible that the CAEP mailing list over- or under-represents the true population of interest. Our demographics and results parallel existing general population trends^30^ and Canadian reports on OSM use by physicians.^2^ Additionally, the demographics of our staff group of respondents are similar to those reported by the CMA.^31^ Our trainee data comes from a single academic center. However, our medical school and residency training programs are the largest in Canada and trainees come from across the world and should represent a diversity of backgrounds and perspectives. While the survey was anonymous, since survey data is self-reported, the prevalence found may not represent the true prevalence of this practice.

Lastly, the response rate was lower than intended, even after numerous reminders, likely due to “survey fatigue” and a long survey (25 items) among physicians.^32^ Nonetheless, the response rate is higher than that of previously published surveys on physician use of social media.^1^,^2^ Additionally, the nature of survey studies is that results are self-reported and often difficult to validate, unless more complexe and resource-intensivemethods are employed. Given all our limitations, compared with previous studies,^1^,^2^ ours is still strengthened by a good response rate from a representative population in an ED clinical setting.

## CONCLUSION

Our findings suggest that although some emergency physicians and trainees use OSM and Google to research their patients, many consider it unethical. Furthermore, we found no age or training-level differences in the likelihood of using Facebook to research patients. Future work should focus on exploring patients’ views on whether this act is desirable and permissible. Research should also explore the nature and content of online searches performed on patients, and the usefulness of information obtained. As the prevalence of social media use by patients increases steadily, individuals, institutions, and professional bodies will need to ethically integrate social media into patient care and medical education.^33^ Autonomy and beneficence should be guiding principles so that we benefit, rather than harm, our patients and profession.

## Figures and Tables

**Figure:**
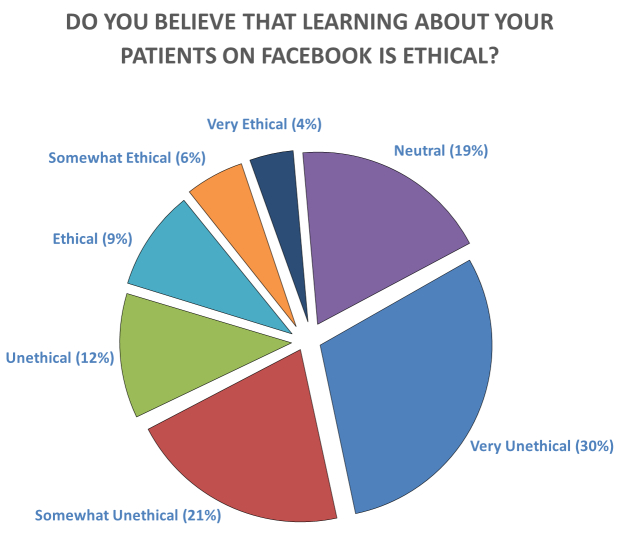
Responses to survey question about ethics of searching for patients online.

**Table 1:** Demographics of survey respondents.

	Medical student (n=185)	Trainee			Staff (n=258)	Total (n=530)

		PGY1–2 (n=46)	PGY3–5 (n=32)	>PGY5/Fellow (n=8)		
Age group, n (%)						
18–24	60 (98.36)	1 (1.63)	0	0	0	61
25–34	122 (51.05)	43 (17.99)	26 (10.88)	6 (2.51)	41 (17.15)	239
35–44	2 (1.57)	2 (1.57)	6 (4.72)	1 (0.79)	116 (91.34)	127
45–54	1 (1.35)	0	0	1 (1.35)	72 (97.30)	74
55+	0	0	0	0	29 (100)	29
Male gender, n (%)	74 (26.24)	19 (6.74)	17 (6.03)	7 (2.48)	164 (58.16)	282
Training program, n (%)						
Emergency medicine	-	31 (44.93)	30 (43.48)	8 (11.59)	-	69
Internal medicine	-	1 (33.33)	2 (66.66)	0	-	3
Family medicine	-	9 (100.00)	0	0	-	9
Surgery	-	3 (100.00)	0	0	-	3
Anesthesiology	-	2 (100)	0	0	-	2

*PGY,* postgraduate year

**Table 2:** Electronic record and social media use among survey respondents.

	Medical student (n=185)	Trainee			Staff (n=258)	Total (n=530)

		PGY1–2 (n=46)	PGY3–5 (n=32)	>PGY5/Fellow (n=8)		
Facebook account, n (%)	170 (43.37)	41 (10.46)	29 (7.40)	7 (1.79)	144 (36.73)	392
Twitter account, n (%)	32 (31.37)	10 (9.80)	5 (4.90)	4 (3.92)	50 (49.02)	102
LinkedIn account, n (%)	27 (30.68)	5 (5.68)	5 (5.68)	2 (2.27)	49 (55.68)	88
Personal blog, n (%)	43 (43.88)	13 (13.27)	5 (5.10)	2 (2.04)	35 (35.71)	98
EMR use in past year, n (%)	95 (26.46)	39 (10.86)	26 (7.24)	6 (1.67)	193 (53.76)	359
Used Facebook to research patients, n (%)	4 (40)	1 (10)	0	2 (20)	3 (30)	10
Used Google to research patients, n (%)	11 (17.19)	9 (14.06)	6 (9.38)	2 (3.13)	36 (56.25)	64

*EMR*, electronic medical record

**Table 3:** Crude and multivariable-adjusted odds ratios of baseline variables for use of Facebook to research patients.

Variable	Crude odds ratio (95% CI)	Multivariable-adjusted odds ratio (95% CI)
Staff	0.39 (0.1–1.5)	0.13 (0.02–1.01)
Age		
25–34	1.97 (0.55–7.08)	1 (reference age class)
35–44	1.27 (0.32–4.98)	4.57 (0.60–34.78)
45–54	0.61 (0.08–4.92)	2.89 (0.18–46.03)
55+	1.85 (0.72–4.71)	1.12 (0.27–4.7)
Male gender	2.01 (0.51–7.85)	1.77 (0.43–7.39)
Use of electronic patient medical record in past year	2.29 (1.10–4.79)	1 (0.2–4.99)
Respondents who are neutral or believe that using Facebook to research patients is ethical	11 (1.41–89.35)	10.37 (1.28–84.15)

**Table 4:** Crude and multivariable-adjusted odds ratios of baseline variables for use of Google to research patients.

Variable	Crude odds ratio (95% CI)	Multivariable-adjusted odds ratio (95% CI)
Staff	0.39 (0.1–1.5)	1.09 (0.42–2.81)
Age		
18–34	0.78 (0.46–1.33)	1 (reference age class)
35–44	1.29 (0.72–2.30)	0.99 (0.37–2.67)
45+	1.07 (0.56–2.02)	0.88 (0.30–2.61)
Male gender	2.04 (1.16–3.60)	1.75 (0.94–3.26)
Use of electronic patient medical record in past year	2.89 (1.28–6.52)	2.12 (0.91–4.90)
Respondents who are neutral or believe that using Google to research patients is ethical	2.93 (1.66–5.16)	2.64 (1.48–4.70)

## References

[1] Bosslet GT, Torke AM, Hickman SE (2011). The patient-doctor relationship and online social networks: results of a national survey. J Gen Intern Med.

[2] Canadian Medical Association (CMA) (2011). Social Media use by physicians: e-Panel Survey Summary.

[3] Thompson LA, Dawson K, Ferdig R (2008). The intersection of online social networking with medical professionalism. J Gen Intern Med.

[4] Warraich HJ (2014). When Doctors ‘Google’ Their Patients, in Well. The New York Times.

[5] Volpe R, Blackall G, Green M (2013). Case study. Googling a patient. Commentary. Hastings Cent Rep.

[6] Mostaghimi A, Crotty BH (2011). Professionalism in the digital age. Ann Intern Med.

[7] Odom-Forren J (2010). Technology: Facebook, Tweets, and the medical record. J Perianesth Nurs.

[8] Gabbard GO, Kassaw KA, Perez-Garcia G (2011). Professional boundaries in the era of the Internet. Acad Psychiatry.

[9] Graham DL (2011). Social media and oncology: Opportunity with risk. American Society for Clinical Oncology Education Book.

[10] O’Hanlon S, Shannon B (2011). Comments further to: Privacy, professionalism and Facebook: a dilemma for young doctors. Med Educ.

[11] Chretien KC, Kind T (2013). Social media and clinical care: ethical, professional, and social implications. Circulation.

[12] Farnan JM, Snyder Sulmasy L, Worster BK (2013). Online medical professionalism: patient and public relationships: policy statement from the American College of Physicians and the Federation of State Medical Boards. Ann Intern Med.

[13] College of Physicians and Surgeons of Ontario (2013). Social Media Terms of Use.

[14] Dillman DA (2007). Mail and internet surveys : the tailored design method.

[15] Mehta N (2012). The New Doctor-Patient Social Media Contract.

[16] Jent JF, Eaton CK, Merrick MT (2011). The decision to access patient information from a social media site: what would you do?. J Adolesc Health.

[17] Ben-Yakov M, Snider C (2011). How Facebook saved our day!. Acad Emerg Med.

[18] Cain J (2008). Online social networking issues within academia and pharmacy education. Am J Pharm Educ.

[19] Gorrindo T, Gorrindo PC, Groves JE (2008). Intersection of online social networking with medical professionalism: can medicine police the facebook boom?. J Gen Intern Med.

[20] McGee JB, Begg M (2008). What medical educators need to know about “Web 2.0”. Med Teach.

[21] Raacke J, Bonds-Raacke J (2008). MySpace and Facebook: applying the uses and gratifications theory to exploring friend-networking sites. Cyberpsychol Behav.

[22] Schleyer T, Spallek H, Butler BS (2008). Facebook for scientists: requirements and services for optimizing how scientific collaborations are established. J Med Internet Res.

[23] Alter AL, Oppenheimer DM (2009). Oppenheimer, Suppressing secrecy through metacognitive ease: cognitive fluency encourages self-disclosure. Psychol Sci.

[24] Cain J, Scott DR, Akers P (2009). Pharmacy students’ Facebook activity and opinions regarding accountability and e-professionalism. Am J Pharm Educ.

[25] Chretien KC, Greysen SR, Chretien JP (2009). Online posting of unprofessional content by medical students. JAMA.

[26] College of Physicians and Surgeons of British Columbia (2010). Social Media and Online Networking Forums.

[27] College Of Physicians And Surgeons Of New Brunswick (2010). Facebook.

[28] Canadian Medical Protective Association (CMPA) (2010). Using social or professional networking websites can breach confidentiality.

[29] American Medical Aassociation (AMA), A.M. Association (2010). Professionalism in the use of social media.

[30] www.socialbakers.com (2012). Canada Facebook Statistics.

[31] Canadian Medical Association (CMA) (2007). Number of physicians by speciality and age.

[32] Couper MP (2007). Issues of Representation in eHealth Research (with a Focus on Web Surveys). Am J Prev Med.

[33] Mesko B (2012). The Social Media course.

